# Sex-Associated Aspects of Referral, Resectability, and Survival in Patients with Colorectal Liver Metastases (CRLM): A Population-Based Single-Center Study of 1207 MDT-Referred Patients

**DOI:** 10.1245/s10434-025-18745-0

**Published:** 2025-11-29

**Authors:** Martina Nebbia, Christina Villard, Stefan Gilg, Ernesto Sparrelid, Marco Gerling, Jennie Engstrand

**Affiliations:** 1 Division of Surgery and Oncology, Department of Clinical Science, Intervention and Technology, Karolinska Institutet, Karolinska University Hospital, Stockholm, Sweden; 2 Division of Transplantation Surgery, Department of Clinical Science, Innovation and Technology, Karolinska Institutet, Stockholm, Sweden

**Keywords:** Colorectal liver metastases (CRLM), Sex differences, KRAS, Liver resection, Ablation, Multidisciplinary team (MDT)

## Abstract

**Background:**

Sex-related differences in colorectal cancer biology and care are increasingly recognized, but their impact on colorectal liver metastases (CRLM) management remains unclear. Previous Swedish registry studies suggested that women undergo CRLM surgery less often than men. This study aimed to further explore sex-associated patterns of referral rates, resectability assessment, and survival in a contemporary, multidisciplinary team (MDT)-reviewed cohort of patients with CRLM.

**Methods:**

The study enrolled all patients with CRLM referred to the Stockholm regional liver MDT between 2013 and 2021. Tumor characteristics, treatment allocation, and survival were compared by sex. Multivariable logistic and Cox regression models were used to assess predictors of resection and survival.

**Results:**

Of 1207 MDT-referred patients, 467 (39%) were women and 740 (61%) were men. The women were younger at diagnosis and had a higher prevalence of *KRAS* mutations, whereas metastatic burden and extrahepatic spread were comparable between the sexes. Curative interventions were performed for 59% of the women and 62% of the men (*p* = 0.219), with no differences in surgical extent, complications, or postoperative outcomes. Among palliatively treated patients, survival was similar, and multivariable Cox models identified no independent association between sex and survival in any subgroup.

**Conclusions:**

In this large, population-based MDT cohort, sex was not associated with differences in resectability, treatment, or survival. The study underscored the importance of centralized and mandatory MDT referral pathways to ensure equitable access to curative-intent treatment and standardized management for all patients with metastatic colorectal cancer.

Colorectal cancer (CRC) is the third most common cancer and the second leading cause of cancer-related death worldwide.^1^ Population-based studies have shown that liver metastases are diagnosed in up to 25 to 30% of patients with CRC, as either a synchronous or metachronous presentation.^2-4^ The management of colorectal liver metastases (CRLM) has evolved substantially during the past two decades, with improved systemic therapies, more complex surgical strategies, and multidisciplinary team (MDT)-based decision-making contributing to better outcomes.^2^^,^^5-8^ Recent evidence further supports the critical role of MDT assessment, showing that evaluation by a liver-focused MDT significantly improves treatment allocation to curative intent and overall survival for patients who have synchronous CRLM, with no sex-based differences observed.^9^

Sex-based differences in CRC epidemiology, tumor biology, and outcomes have been increasingly recognized: women have CRC diagnosed at an older age, present more frequently with right-sided tumors, and have different molecular profiles, including a higher prevalence of BRAF mutations and microsatellite instability.^10-12^ These factors may influence disease presentation and treatment response. In early-stage CRC, female sex has been associated with better survival, but findings in the metastatic setting are inconsistent.^9^^,^^13-15^

A recent Swedish national registry study raised concerns about lower resection rates among women with CRLM.^6^ One possible explanation is that women may present with more extensive or extrahepatic disease. Another is that they might be more likely to decline surgical treatment. These concerns reflect broader patterns observed in the metastatic setting, in which access to surgical management varies. Several studies have shown that women with liver metastases may be less likely to be referred for or undergo hepatic resection, potentially due to age, comorbidity, or differences in clinical presentation.^16-18^

**Fig. 1:**
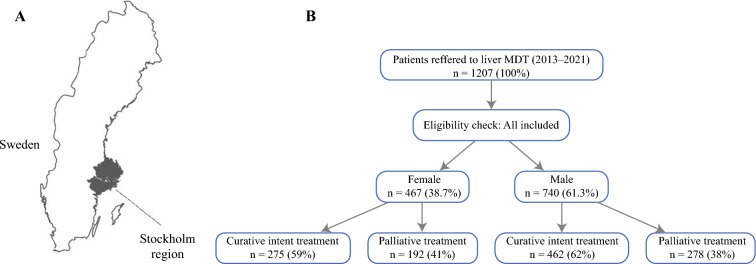
**A** Map of Sweden with the population-derived cohort defined from the Stockholm region. **B** Study flowchart of patients with colorectal liver metastases referred to the regional liver MDT between 2013 and 2021. MDT, multidisciplinary team

Accurate assessment of resectability is a cornerstone in the management of CRLM. Multiple population-based and institutional studies have shown that specialized hepatobiliary multidisciplinary teams, with direct involvement of experienced liver surgeons, lead to more accurate assessments of resectability and broader access to curative-intent surgery.^19-24^ Such expertise has been associated with higher resection rates, more standardized treatment strategies, and improved survival outcomes.^25^ However, evidence remains inconsistent, and few investigations have comprehensively evaluated sex-specific differences in CRLM using large, real-world cohorts subjected to standardized MDT review.

In this study, we aimed to assess sex-related patterns of referral, resectability, and survival in a cohort of patients with CRLM referred to a tertiary liver MDT. By analyzing sex-specific patterns in CRLM management and outcomes, our data highlight current treatment strategies and identify systematic MDT referral as a key means of ensuring equitable care for both women and men with metastatic CRC.

## Methods

### Study Design and Population

In this population-based cohort study, all patients with CRLM referred to the regional liver MDT between 1 January 2013 and 31 December 2021 were retrospectively identified. The Stockholm health care region serves a population of approximately 2.4 million and has a centralized hepatobiliary surgical service located exclusively at Karolinska University Hospital (Fig.1).

**Fig. 2:**
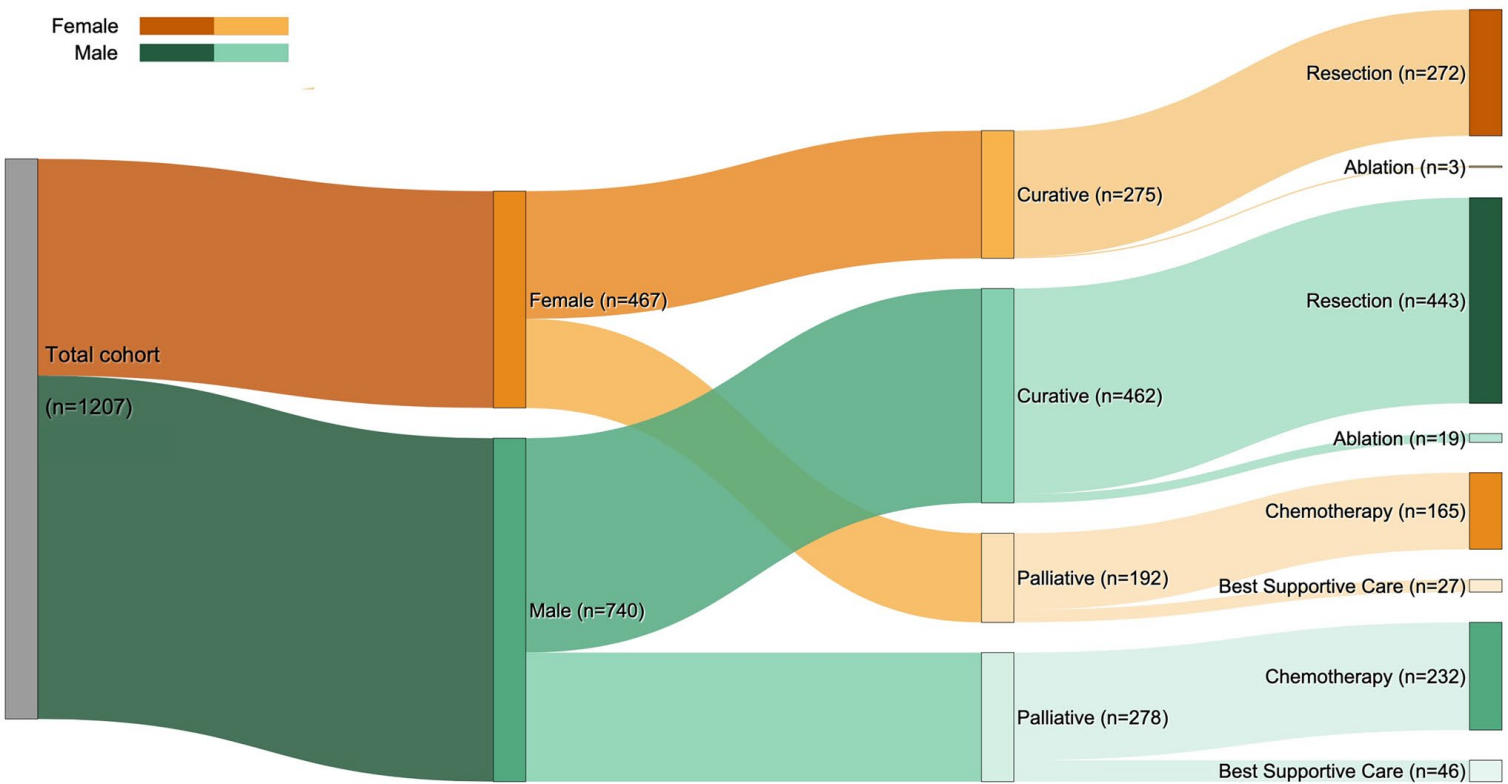
Sankey diagram of treatment allocation for 1207 patients with colorectal liver metastases referred to the regional liver MDT (2013–2021), stratified by gender

All patients with histologically confirmed CRC and radiologically suspected or confirmed liver metastases on contrast-enhanced computed tomography and/or magnetic resonance imaging referred to the liver MDT were eligible (Fig 2). There were no age or performance status restrictions for liver MDT referral, nor were patients with suspected or confirmed extrahepatic metastases excluded from the study cohort. Each case was evaluated by a multidisciplinary team including hepatobiliary surgeons, a transplant surgeon, radiologists, a hepatologist, oncologists, and a colorectal surgeon if it involved synchronous presentation of liver metastases.

### Data Collection

Clinical, pathologic, and treatment data were retrieved from electronic medical health records. The variables collected were age, biologically assigned sex, Eastern Cooperative Oncology Group performance status, American Society of Anesthesiologists score, body mass index, primary tumor site, tumor and nodal status, carcinoembryonic antigen (CEA) level, liver metastasis tumor burden (number, size, bilobar involvement), synchronous versus metachronous presentation, ^26^ and extrahepatic disease. When available, KRAS proto-oncogene, GTPase (KRAS) and B-Raf proto-oncogene, and serine/threonine kinase (BRAF) mutational status were recorded. For each patient, MDT recommendations, treatment allocation, extent of liver resection, and if no resection occurred, the reasons for non-surgery (e.g., progression on chemotherapy, non-resectability, and non-treatable extrahepatic disease or patient refusal) were documented. Ethical approval was granted by the National Ethical Review Board, Sweden, #2019-01571, and amendments 2020-00428, 2021-06863-02, 2022-06588-02, and 2025-05423-02.

### Statistical Analysis

Continuous variables were summarized using medians and interquartile ranges (IQRs) and compared using the Mann–Whitney U test. Categorical variables were compared using chi-square or Fisher’s exact tests. Factors associated with receipt of liver intervention were analyzed using logistic regression and presented using odds ratios (ORs) with 95% confidence intervals (CIs). Survival time was calculated from the date of liver MDT for the whole study cohort and for the palliatively treated subgroup, and from the date of liver intervention for the resected subgroup. Survival curves were estimated using the Kaplan–Meier method and compared with the log-rank test. Cox proportional hazards regression was used to identify factors independently associated with survival, and results were presented as hazard ratios (HRs) with 95% CIs. The variables included in multivariable models were limited to those variables known in both the resected and palliatively treated cohorts, and included age, tumor location, nodal status, number and size of metastases, extrahepatic disease, synchronous detection, year of MDT referral, and sex. Adjusted odds ratios (aORs) and hazard ratios (aHRs) were reported with 95% CIs.

Statistical significance was set at a p value lower than 0.05. All analyses were performed using STATA/MP 18.5 (StataCorp, College Station, TX, USA).

## Results

### Sex Aspects of Referral and Presentation

Among 1207 patients with CRLM referred to the regional liver MDT between 2013 and 2021, 467 (39%) were women and 740 (61%) were men. The women were slightly younger at the time of the CRLM diagnosis than the men (median age, 65 years [IQR, 54–73 years] vs 68 years [IQR, 60–74 years]; *p *< 0.001) and had a higher prevalence of KRAS mutations (55 vs 42%; *p* = 0.003), based on the 567 patients (47%) with available molecular testing. The BRAF mutation rates were similar between the sexes (13% for the women vs 10% for the men; *p* = 0.338). In the more recent study period, the proportion of female patients increased from 35% in 2013–2016 to 41% in 2017–2021 (*p* = 0.037; Table 1).

**Table 1:** Baseline characteristics of 1207 patients with colorectal liver metastases (CRLM) referred to the regional liver MDT between 2013 and 2021

Variables	Overall (*n *= 1207) *n* (%)	Female (*n *= 467) *n* (%)	Male (*n *= 740) *n* (%)	*p* Value^a^
Median age at diagnosis: years (IQR)	67 (56–74)	65 (54–73)	68 (60–74)	**<0.001**
*Year of MDT discussion*				**0.037**
2013–2016	578 (48)	206 (35)	372 (64)	
2017–2021	629 (52)	261 (41)	368 (58)	
*Primary tumor*				
Right-sided colon	370 (31)	155 (35)	215 (29)	0.087
Left-sided colon	423 (36)	162 (36)	261 (36)	
Rectum	387 (32)	132 (29)	255 (35)	
Positive nodal status	727 (74)	284 (76)	443 (72)	0.219
Median CEA at diagnosis: µg/L (IQR)	11.5 (3.3–64)	12 (3.4–53)	11 (3.2–78)	0.832
Primary tumor resected	747 (62)	278 (61)	469 (64)	0.254
*Liver metastases*				
1	324 (27)	119 (25)	205 (28)	0.160
2–5	372 (31)	160 (34)	212 (29)	
6–10	199 (16)	68 (15)	131 (18)	
>10	312 (26)	120 (26)	192 (26)	
Median size of largest metastasis: mm (IQR)	28 (17–47)	26 (16–45)	30 (17–48)	0.178
Bilobar metastases	707 (59)	279 (60)	428 (58)	0.530
Synchronous detection	792 (66)	305 (65)	487 (66)	0.859
Extrahepatic metastases	328 (27)	138 (30)	190 (26)	0.141
Lung metastases	266 (22)	114 (24)	152 (21)	0.117
Liver resection	737 (61)	275 (59)	462 (62)	0.219
Molecular profile				
RAS/BRAF tested	559 (46)	222 (48)	337 (46)	0.456
*KRAS* mutant	263 (47)	122 (55)	141 (42)	**0.003**
*BRAF* mutant	62 (11)	28 (13)	34 (10)	0.338

*MDT* multidisciplinary team; *IQR* interquartile range; *CEA* carcinoembryonic antigen; *RAS* rat sarcoma; *BRAF* V-Raf murine sarcoma viral oncogene homolog B; *KRAS* Kirsten rat sarcoma viral oncogene homolog

^a^*p* Values compare male and female; bold values indicate statistical significance (*p *< 0.05)

The distribution of nodal status, number and size of CRLM, bilobar involvement, and presence of extrahepatic disease did not differ significantly between the sexes. Right-sided colon cancers were more frequent among the women (35 vs 29%), whereas rectal primaries were more common among the men (35 vs 29%), although these differences were not statistically significant (*p* = 0.087).

The extent of liver disease at presentation was substantial in both sexes: approximately one fourth of the patients had more than 10 CRLMs, and bilobar involvement was present in 60% of the women and 58% of the men (*p* = 0.530). The rates of synchronous detection were nearly identical (65% for the women vs 66% for the men; *p* = 0.859), and extrahepatic disease was observed in 30% of the women and 26% of the men (*p* = 0.141), with lung the most frequent site of metastases (24 vs 21%; *p* = 0.117).

### Resectability and Treatment Allocation

Overall, 737 patients (61%) underwent liver resection or ablation, whereas 470 (39%) were managed palliatively. Liver intervention rates did not differ significantly by sex (59% for the women vs 62% for the men; *p *= 0.219).

The proportion of women among all the patients undergoing resection increased significantly over time, from 33% in 2013–2016 to 41% in 2017–2021 (*p *= 0.017). However, when resection rates were analyzed relative to all the women referred to the MDT, no significant change was observed (56 vs 61%; *p *= 0.360).

Further liver intervention characteristics are presented in Table 2.

**Table 2:** Patient, metastases, and intervention characteristics in 737 patients with CRLM undergoing resection or ablation between 2013 and 2021

Variables	Overall (*n *= 737) *n* (%)	Female (*n *= 275) *n* (%)	Male (*n *= 462) *n* (%)	*p* Value^a^
Median age at diagnosis: years (IQR)	67 (59–74)	66 (56–73)	68 (61–74)	**0.016**
*Year of MDT discussion*				
2013–2016	353 (48)	116 (33)	237 (41)	**0.017**
2017–2021	384 (52)	159 (41)	225 (59)	
*ASA*				
1–2	384 (52)	156 (57)	228 (49)	**0.046**
3–4	352 (48)	118 (43)	234 (51)	
*ECOG*				
0	582 (79)	215 (78)	367 (79)	0.952
1	133 (18)	51 (19)	82 (18)	
2–3	21 (3)	8 (3)	13 (3)	
Median BMI: kg/m^2^ (IQR)	25 (23–28)	24 (22–27)	26 (24–28)	**<0.001**
*Primary tumor*				
Right-sided colon	195 (26)	81 (30)	114 (25)	0.223
Left-sided colon	270 (37)	102 (37)	168 (37)	
Rectum	267 (36)	90 (33)	177 (39)	
Positive nodal status	460 (70)	180 (73)	280 (68)	0.156
Median CEA at diagnosis: µg/L (IQR)	6.7 (2.5–29.0)	6.8 (2.4–28.0)	6.7 (2.8–29)	0.898
Primary tumor resected	677 (92)	250 (94)	427 (93)	0.770
RAS/BRAF tested	347 (47)	136 (50)	211 (46)	0.277
*KRAS* mutant^b^	168 (48)	80 (58)	88 (42)	**0.003**
*BRAF* mutant^b^	27 (8)	13 (10)	14 (7)	0.305
*Metastases*				
1	279 (38)	101 (37)	178 (38)	**0.040**
2–5	299 (41)	127 (46)	172 (37)	
6–10	100 (14)	32 (12)	68 (15)	
>10	59 (8)	15 (5)	44 (10)	
Median size of largest metastasis: mm (IQR)	24 (15–39)	22 (15–38)	25 (15–40)	0.233
Bilobar metastases	292 (40)	106 (39)	186 (40)	0.629
Synchronous detection	386 (52)	138 (50)	248 (54)	0.358
Simultaneous extrahepatic metastases	126 (17)	53 (19)	73 (16)	0.226
Lung metastases	89 (12)	38 (14)	51 (11)	0.267
Non-pulmonary metastases	43 (6)	20 (7)	23 (5)	0.199
Chemotherapy				
Neoadjuvant	447 (61)	177 (65)	270 (59)	0.102
Adjuvant	350 (52)	137 (56)	213 (51)	0.291
*Liver intervention characteristics*				
PVE	38 (5)	10 (4)	28 (6)	0.148
*Extent of first liver resection*				
Minor	419 (57)	154 (56)	265 (57)	**0.041**
Major	296 (40)	118 (43)	178 (39)	
Thermal ablation	22 (3)	3 (1)	19 (4)	
Simultaneous primary tumor resection	127 (17)	50 (18)	77 (17)	0.598
Median intraoperative bleeding: mL (IQR)	400 (200–800)	300 (150–600)	500 (200–900)	**<0.001**
Median hospital stay: days (IQR)	8 (6–10)	8 (6–10)	8 (6–11)	0.211
Major complications	147 (20)	56 (20)	91 (20)	0.838

*CRLM* colorectal liver metastases; *IQR* interquartile range; *MDT* multidisciplinary team; *ASA* American Society of Anesthesiologists’ classification system; *ECOG* Eastern Cooperative Oncology Group performance status; *BMI* body mass index; *CEA* carcinoembryonic antigen; *RAS* rat sarcoma; *BRAF* V-Raf murine sarcoma viral oncogene homolog B; *KRAS* Kirsten rat sarcoma viral oncogene homolog; *PVE* portal vein embolization

^a^*p* Values compare male and. female. Bold values indicate statistical significance (*p *< 0.05)

^b^Percentage among those tested

Multivariable logistic regression showed no independent association between sex and the likelihood of liver intervention (aOR, 1.19; 95% CI, 0.76–1.85; *p *= 0.450). Furthermore, in the multivariable model, the patients older than 80 years and those with multiple CRLMs, synchronous presentation, and extrahepatic disease were significantly less likely to undergo a liver intervention (Table 3). The later MDT referral period (2017–2021) was associated with a higher likelihood of resection compared with 2013–2016 (aOR 2.42; 95% CI 1.57–3.75).

**Table 3:** Factors associated with liver intervention in 1207 patients referred to liver MDT between 2013 and 2021

Variable	Univariable analysis			Multivariable analysis		
	OR	95% CI	*p* Value^a^	OR	95% CI	*p* Value^a^
Male gender	1.16	0.92–1.47	0.219	1.19	0.76–1.85	0.450
*Age (years)*						
<50	Reference category^b^			Reference category^b^		
51–60	1.08	0.71–1.65	0.713	1.26	0.60–2.64	0.534
61–70	1.37	0.93–2.02	0.112	0.72	0.37–1.43	0.353
71–80	1.32	0.90–1.96	0.159	0.53	0.26–1.05	0.070
>80	0.70	0.41–1.22	0.209	0.13	0.05–0.33	**<0.001**
*Year of MDT discussion*						
2013–2016	Reference category			Reference category		
2017–2021	0.99	0.79–1.26	0.993	2.42	1.57–3.75	**<0.001**
*Primary tumor location*						
Right colon	Reference category			Reference category		
Left colon	1.58	1.19–2.11	**0.002**	1.20	0.73–1.99	0.475
Rectum	2.00	1.48–2.69	**<0.001**	1.96	1.17–3.28	**0.011**
Positive nodal status	0.50	0.36–0.69	**<0.001**	0.55	0.34–0.90	**0.018**
CEA >10	0.38	0.28–0.52	**<0.001**	0.93	0.59–1.44	0.738
Primary tumor resected	83.7	56.5–123.8	**<0.001**			
*No. of liver metastases*						
1	Reference category			Reference category		
2–5	0.66	0.44–0.99	**0.045**	1.01	0.56–1.84	0.970
6–10	0.16	0.11–0.25	**<0.001**	0.18	0.10–0.33	<0.001
>10	0.04	0.02–0.06	**<0.001**	0.05	0.03–0.10	<0.001
Size of largest metastasis >5 cm	0.41	0.31–0.54	**<0.001**	0.69	0.42–1.12	0.131
Bilobar metastases^c^	0.09	0.06–0.12	**<0.001**			
Synchronous detection	0.17	0.13–0.23	**<0.001**	0.23	0.13–0.40	<0.001
Extrahepatic metastases	0.27	0.21–0.36	**<0.001**	0.25	0.16–0.41	<0.001

*MDT* multidisciplinary team; *OR* odds ratio; *CI* confidence interval; *CEA* carcinoembryonic antigen

^a^Bold *p* values indicate statistical significance (*p *< 0.05)

^b^Reference category indicates baseline group for categorical variables

^c^Bilobar metastases excluded from multivariable model due to collinearity with number of metastases

### Characteristics of Palliatively Treated Patients

Among the 470 patients managed without curative-intent liver treatment, 192 (41%) were female and 278 (59%) were male. The women in this subgroup were significantly younger at diagnosis than the men (median, 65 vs 68 years; *p *= 0.002), consistent with the trend observed in the overall cohort. The distribution of the MDT referral period (2013–2016 vs 2017–2021) was comparable between the sexes (Table 4).

**Table 4:** Patient and metastases characteristics of 470 palliatively treated patients with colorectal liver metastases referred to liver MDT between 2013 and 2021

Variables	Overall (*n* = 470) *n* (%)	Female (*n* = 192) *n* (%)	Male (*n* = 278) *n* (%)	*p* Value
Median age at diagnosis: years (IQR)	67 (56–74)	65 (52–74)	68 (60–74)	
*Year of MDT discussion*				
2013–2016	225 (48%)	90 (47%)	135 (49%)	0.719
2017–2021	245 (52%)	102 (53%)	143 (51%)	
*Primary tumor*				
Right-sided colon	175 (37%)	74 (42%)	101 (37%)	0.453
Left-sided colon	153 (33%)	60 (34%)	93 (34%)	
Rectum	120 (26%)	42 (24%)	78 (29%)	
Positive nodal status	267 (82%)	104 (82%)	163 (82%)	0.921
Median CEA at diagnosis: µg/L (IQR)	30 (5.5–170)	24 (6–126)	37 (5–219)	0.595
Primary tumor resected	70 (15%)	28 (15%)	42 (15%)	0.850
*Liver metastases*				
1	45 (10%)	18 (9%)	27 (10%)	0.693
2–5	73 (16%)	33 (17%)	40 (14%)	
6–10	99 (21%)	36 (19%)	63 (23%)	
>10	253 (54%)	105 (55%)	148 (53%)	
Median size of largest metastasis: mm (IQR)	36 (20–61)	30 (20–55)	40 (20–65)	0.113
Bilobar metastases	415 (88%)	173 (90%)	242 (87%)	0.311
Synchronous detection	406 (86%)	167 (87%)	239 (86%)	0.754
Extrahepatic metastases	202 (43%)	85 (44%)	117 (42%)	0.638
Lung metastases	177 (38%)	76 (40%)	101 (36%)	0.474
RAS/BRAF mutational status tested	212 (45%)	86 (45%)	126 (45%)	0.909
*KRAS* mutant	95 (45%)	42 (49%)	53 (42%)	0.330
*BRAF* mutant	35 (16%)	15 (17%)	20 (16%)	0.763
Palliative chemotherapy	397 (84%)	165 (86%)	232 (83%)	0.465

*MDT* multidisciplinary team; *IQR* interquartile range; *CEA* carcinoembryonic antigen; *RAS* rat sarcoma; *BRAF* V-Raf murine sarcoma viral oncogene homolog B; *KRAS* Kirsten rat sarcoma viral oncogene homolog

Tumor location, nodal involvement, CEA levels, number and distribution of CRLMs, and presence of extrahepatic disease all were similar between the female and male patients. Bilobar involvement was present in nearly 90% of both sexes, and more than 10 CRLMs were observed in 55% of the women and 53% of the men.

In addition, the rates of synchronous detection (87 vs 86%), primary tumor resection (15% for both sexes), and molecular testing (45% for both sexes) were almost identical for the men and women.

Among the patients with molecular testing, KRAS mutations were slightly more common among the women (49 vs. 42%), and BRAF mutations were more common among the men (17 vs 16%), although these differences did not reach statistical significance (*p* = 0.405 vs 0.909). Use of palliative chemotherapy was similar across the sexes (86% of the women vs 83% of the men; *p* = 0.465).

### Factors Associated With Overall Survival

**Fig. 3:**
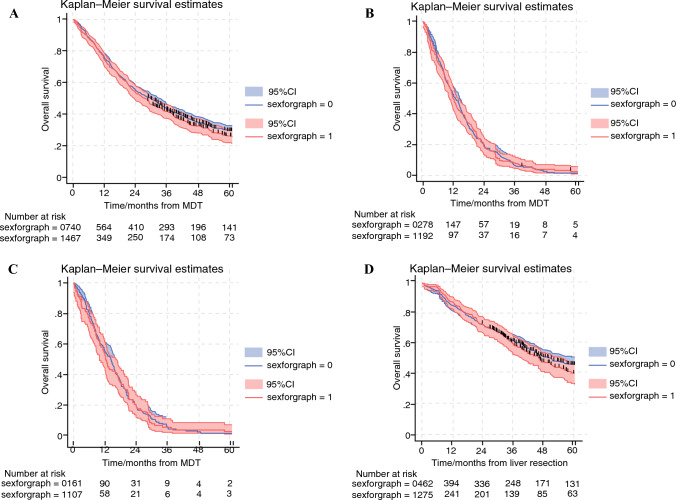
Overall survival stratified by sex in patients with colorectal liver metastases (CRLM) referred to the liver MDT between 2013 and 2021. **A** All referred patients (*n *= 1207). Median survival: 29.5 months for males vs 27.5 months for females; 5-year OS: 29.4 vs 26.2% (*p *= 0.286, log-rank). Female sex was not associated with survival (aHR, 0.96; 95% CI, 0.82–1.13). **B** Palliatively treated patients (*n *= 470). Median survival: 29.5 months for males vs 27.5 months for females; 5-year OS, 29.4 vs 26.2% (*p *= 0.943, log-rank). Female sex was not associated with survival (aHR, 0.99; 95% CI, 0.81–1.23). **C** Palliatively treated patients with liver-only disease (*n *= 268). Median survival was similar between the sexes (*p *= 0.819, log-rank). Female sex was not associated with survival (aHR, 0.97; 95% CI, 0.76–1.24). **D** Patients undergoing liver resection or ablation (*n *= 737). Median survival: 48 months for males vs 49 months for females; 5-year OS, 39.5 vs 42.0%; (*p *= 0.546, log-rank). Female sex was not associated with survival (aHR, 0.96; 95% CI, 0.76–1.22). MDT, multidisciplinary team; OS, overall survival; aHR, ; CI, confidence interval

In the full cohort of 1207 patients, the median survival was 29.5 months for the men and 27.5 months for the women (*p* = 0.286, log-rank). The 5-year overall survival (OS) was 29.4% (95% CI, 25.9–33.0%) for the men and 26.2% (95% CI, 21.9–30.7%) for the women (Fig. 3A). In the univariable Cox regression, sex was not associated with survival, and this finding remained non-significant in the multivariable model (aHR, 0.93; 95% CI, 0.76–1.14; *p* = 0.460) after adjustment for age, tumor location, nodal status, number and size of liver metastases, synchronous detection, and extrahepatic disease) (Table 5).

**Table 5:** Factors associated with survival in 1207 patients referred to liver MDT between 2013 and 2021

Variable	Univariable analysis			Multivariable analysis		
	HR	95% CI	*p* Value^a^	HR	95% CI	*p* Value^a^
Male gender	0.93	0.81–1.06	0.286	0.93	0.76–1.14	0.460
*Age (years)*						
<50	Reference category^b^			Reference category^b^		
51–60	0.82	0.64–1.05	0.117	0.71	0.51–0.99	**0.045**
61–70	0.86	0.69–1.07	0.178	1.02	0.75–1.40	0.878
71–80	1.07	0.85–1.33	0.565	1.56	1.14–2.13	**0.006**
>80	1.47	1.09–1.99	**0.012**	3.04	1.70–4.69	**<0.001**
*Year of MDT discussion*						
2013–2016	Reference category					
2017–2021	1.08	0.94–1.23	0.298			
*Primary tumor location*						
Right colon	Reference category			Reference category		
Left colon	0.65	0.55–0.76	**<0.001**	0.74	0.59–0.94	**0.012**
Rectum	0.64	0.54–0.75	**<0.001**	0.64	0.50–0.80	**<0.001**
Positive nodal status	1.92	1.59–2.32	**<0.001**	1.68	1.32–2.14	**<0.001**
CEA >10 µg/L	1.64	1.38–1.95	**<0.001**	1.14	0.92–1.41	0.223
*No. of liver metastases*						
1	Reference category			Reference category		
2–5	1.42	1.16–1.72	**0.001**	1.34	1.02–1.78	**0.039**
6–10	2.67	2.15–3.31	**<0.001**	3.30	2.45–4.45	**<0.001**
>10	4.08	3.37–4.95	**<0.001**	4.00	2.94–5.42	**<0.001**
Size of largest metastasis >5 cm	1.58	1.36–1.84	**<0.001**	1.36	1.09–1.70	**0.006**
Bilobar metastases^c^	2.71	2.35–3.14	**<0.001**			
Synchronous detection	1.52	1.32–1.76	**<0.001**	1.06	0.84–1.34	0.639
Extrahepatic metastases	2.06	1.79–2.38	**<0.001**	1.87	1.53–2.28	**<0.001**

*MDT* multidisciplinary team; *HR* hazard ratio; *CI* confidence interval; *CEA* carcinoembryonic antigen

^a^Bold *p* values indicate statistical significance (*p *< 0.05)

^b^Reference category indicates baseline group for categorical variables

^c^Bilobar metastases excluded from multivariable model due to collinearity with number of metastases

Among the palliatively treated patients (*n* = 470), survival was similarly equivalent between the sexes, with a median survival of 27.5 months for the women and 29.5 months for the men, and a 5-year OS of 26.2% for the women and 29.4% for the men (*p* = 0.943; Fig. 3B). In a separate model (data not shown) limited to the palliatively treated subgroup (*n* = 470), sex remained non-significant (aHR, 0.99; 95% CI, 0.81–1.23; *p* = 0.993), reinforcing the finding that female sex is not associated with survival outcomes for patients with advanced CRLM referred to liver MDT. In the subgroup of patients who had liver-only metastases managed with palliative intent (*n* = 268), there was no significant survival difference between the sexes (*p* = 0.819, log-rank; univariable HR, 0.97; 95% CI, 0.76–1.24; Fig. 3C).

Among the patients undergoing liver resection or ablation (n = 737), the survival outcomes were similar between the sexes, with a median survival of 49 months for the women and 48 months for the men, and a 5-year OS of 42.0% for the women and 39.5% for the men (Fig. 3D; *p* = 0.546, log-rank). In the multivariable Cox regression on factors influencing survival among the resected patients (data now shown), female sex was not associated with survival (aHR, 0.96; 95% CI, 0.76–1.22).

## Discussion

This study provides evidence that patient sex is not an independent predictor of survival for CRLM for patients evaluated and managed through a standardized liver MDT process. In our large, contemporary cohort of 1207 patients discussed at a hepatobiliary MDT during a 9-year period, we observed no significant differences in liver resection rates, survival outcomes, or access to palliative or curative-intent treatment between women and men. These findings held true across all major subgroups, including those undergoing liver resection, those treated palliatively, and those with liver-only metastatic disease. These findings are consistent with recent data from the South-East Health Care Region of Sweden, in which assessment by a liver-focused MDT was independently associated with improved survival and no sex-based differences in treatment access or outcomes were observed.^5^

Our analyses further showed no significant differences in liver resection rates between men and women after adjustment for clinical and tumor-related factors. Although male sex showed a non-significant trend toward greater likelihood of resection in the univariable analysis, this was not maintained in the multivariable model. Importantly, patient age, tumor burden, and extent of disease—not sex—were the dominant determinants of resection eligibility, with markedly decreased resection rates among patients older than 80 years and those with multiple or bilobar metastases, extrahepatic spread, or synchronous presentation. This underscores that in a modern Swedish MDT setting, sex alone does not constitute a barrier to curative-intent liver treatment.

The female patients were, on average, younger at diagnosis and had a significantly greater prevalence of KRAS mutations known to be associated with poorer prognosis and resistance to estimated glomerular filtration rate (EGFR) inhibitors. Nevertheless, this did not translate into a survival disadvantage. Median and 5-year survival rates were comparable between the genders, and multivariable Cox regression models consistently demonstrated that sex was not associated with overall survival in the total cohort or in subgroup analyses. Even after adjustment for potential confounders (age, tumor location, number and size of liver metastases, synchronous detection, and extrahepatic disease), female gender remained a neutral prognostic factor.

These results are consistent with prior population-based studies suggesting that disease biology rather than sex drives prognosis in metastatic CRC (mCRC). For instance, analyses from the Surveillance, Epidemiology, and End Results (SEER) database and large European cancer registries have shown that once access to surgery and systemic therapy is accounted for, survival outcomes between men and women converge.^27-29^ However, some earlier reports raised concerns that women may be under-referred for surgery or receive less aggressive treatment, possibly due to older age at diagnosis or a greater prevalence of right-sided tumors, which often are associated with less favorable biology.^30,31^

In contrast to our findings, which showed no sex-related differences in resectability, treatment allocation, or survival, de Graaff et al.^32^ reported that women with synchronous CRLM were significantly less likely to undergo local treatment despite comparable postoperative outcomes. This discrepancy suggests that centralized referral pathways and structured MDT evaluation, as applied in our cohort, may help to reduce sex-related disparities observed at the population level. Although no sex differences were demonstrated in our study, it remains unknown whether disparities may arise before referral to the liver MDT due to either conscious or unconscious referral patterns by colorectal surgeons/oncologists, or because women more frequently present with truly non-resectable extrahepatic disease. This further underscores the importance of ensuring liver MDT evaluation for all patients with reasonable physiologic reserve and acceptable performance status.

Our findings also should be considered in the context of emerging data on early-onset colorectal cancer, which is increasing in incidence worldwide.^33-35^ The review by Char et al.^36^ highlights that early-onset colorectal cancer often presents with distinct clinic-pathologic and molecular characteristics, including a higher proportion of left-sided or rectal primaries, advanced stage at diagnosis, and a greater prevalence of unfavorable biomarkers such as KRAS and BRAF mutations. These features may influence both the biology of metastatic spread and the response to systemic and liver-directed therapies. Importantly, younger patients, who are more often women in certain cohorts, may have differing comorbidity profiles and treatment tolerability compared with older patients. Although our cohort did not specifically stratify by early-onset status, the increasing referral of women to the MDT in recent years could partially reflect demographic shifts in CRC incidence. Integrating age-of-onset with sex-based analyses in future CRLM research could provide a more nuanced understanding of referral patterns, treatment allocation, and long-term outcomes.

Recent national registry studies, such as that by Ljunggren et al.,^30^ reignited this debate by demonstrating significantly lower resection rates among women with metastatic CRC. The authors proposed two potential explanations: first, that women may present more frequently with unresectable or extrahepatic disease, and second, that women may be more likely to decline metastasectomy. These hypotheses directly informed our investigation into a well-defined MDT-reviewed population from the Stockholm region.

In our cohort, the extent of metastatic disease at presentation did not differ by sex, with comparable distribution regarding number of liver metastases, bilobar involvement, and presence of extrahepatic disease. These findings suggest that when patients are systematically evaluated by a liver MDT, gender-based disparities in resection rates diminish or disappear.^19-24,37^

Assessment in a dedicated liver MDT is crucial because both oncologists and colorectal surgeons have been shown to frequently underestimate hepatic resectability, whereas centralized multidisciplinary review increases resection rates and improves survival.^19-24^

Additionally, temporal analyses showed that in the later part of the study period (2017–2021), the proportion of referred women increased, supporting the idea of improving referral equity over time. Our findings also align with those of Nordenvall et al.^6^ and underscore the importance of centralized MDT referral in improving access and standardizing care. The Stockholm region’s proximity to a university hospital and adherence to MDT protocols may contribute to higher and more equitable resection rates compared with other parts of Sweden. This observation lends weight to the argument that mandatory referral of all patients with CRLM, regardless of disease extent or demographic characteristics, is essential to reduce treatment variability and ensure equal opportunity for curative therapy.

Logistic regression models in our study confirmed that established prognostic variables, such as age older than 80 years, extensive liver disease (>10 metastases), synchronous presentation, and extrahepatic spread, were associated with lower likelihood of resection. Gender was not a contributing factor. Furthermore, although KRAS mutations were significantly more common in women, survival outcomes remained similar. This supports the notion that MDT-based evaluation, which incorporates disease anatomy, response to therapy, and resectability, can neutralize the effect of unfavorable biomarkers on treatment decisions and survival.

Interestingly, contrasting results have been reported in more selective treatment settings. A recent multicenter liver transplantation (LT) cohort study for CRLM found that female sex was independently associated with worse overall survival after LT (multivariable LASSO Cox HR, 4.1; 95% CI, 1.8–9.2).^38^ In that analysis, additional adverse predictors included CEA exceeding 80 µg/L, right-sided primary tumor, largest liver metastasis diameter greater than 5.5 cm, KRAS mutation, and absence of prior liver-directed therapy. These findings highlight that gender-related survival differences may emerge in highly selected therapeutic contexts, such as LT, in which patient selection criteria, tumor biology, and timing of interventions differ markedly from those of standard MDT-guided resection cohorts. The divergence from our results underscores the importance of context when gender effects are interpreted and suggests that the interplay between tumor biology and extreme treatment selection pressures may accentuate differences not apparent in broader clinical practice.

In summary, our study demonstrated that in a structured MDT setting, patients with CRLM receive equitable assessment and treatment irrespective of sex. No differences in resectability, survival, or treatment allocation were observed in any subgroup, including patients with liver-only disease or those treated palliatively. These findings challenge historical concerns of sex disparity and emphasize the need for universal referral pathways to specialized liver MDTs. Importantly, our data suggest that disparities reported in national studies may reflect gaps in referral rather than intrinsic differences in disease biology or treatment response.

### Study Limitations

This study had limitations. First, the retrospective design may have introduced residual confounding despite comprehensive multivariable modeling. Second, molecular profiling (particularly KRAS and BRAF status) was available only for approximately half of the patients, limiting the power of genotype-adjusted analyses. Third, our study was conducted at a high-volume tertiary center with standardized MDT processes, which may not be generalizable to all settings, particularly where access to specialized liver surgery is limited.

## Conclusion

In this population-based study of 1207 patients with colorectal liver metastases discussed at a regional multidisciplinary conference, we found no sex-associated differences in referral patterns, resectability, or survival outcomes. Despite biologic differences, including higher KRAS mutation rates among women, treatment allocation and prognosis were comparable across genders in both curative and palliative settings. Our results suggest that standardized multidisciplinary conference assessment can mitigate previously reported disparities in access to liver resection. These findings underscore the importance of mandatory referral to specialized liver teams to ensure equitable, biology-driven care for all patients.
